# 607. Angiotensin Converting Enzyme Inhibitor Modulation of Angiotensin II and Bradykinin on Burn Patient Outcomes

**DOI:** 10.1093/jbcr/irag033.418

**Published:** 2026-04-06

**Authors:** Juquan Song, Dalton Buckingham, Amina El Ayadi, Steven E Wolf

**Affiliations:** University of Texas Medical Branch, Galveston, TX; University of Texas Medical Branch - John Sealy School of Medicine, Galveston, TX; Division of Burn and Trauma Surgery, Department of Surgery, University of Texas Medical Branch, Galveston, Galveston, TX; Division of Burn and Trauma Surgery, Department of Surgery, University of Texas Medical Branch, Galveston, Galveston, TX

## Abstract

**Introduction:**

Angiotensin converting enzyme inhibitors (ACEi) are one of the most prescribed drugs in the United States, with proven efficacy as an anti-hypertensive as well as an organ protectant in common conditions such as diabetes mellitus, ischemic heart disease, chronic heart failure, and chronic kidney disease through modulation of the renin-angiotensin-aldosterone system.

**Methods:**

We used data from the TriNetX electronic health database to create six cohorts of patients who sustained a burn injury and were above the age of 18. These cohorts were defined by the presence or absence of an ACEi or other common anti-hypertensive – beta blockers, calcium channel blockers, diuretics, or angiotensin receptor blockers (ARBs). We then compared the prevalence of specific adverse effects (AE) – angioedema, infection, pneumonia, sepsis, re-hospitalization, and mortality – between patients administered ACEi, those not, and those on another antihypertensive for three separate time ranges, coinciding with the proposed time of the different immune states following thermal injury: acute (1-14 days), suppressive (14 days-90 days), and resolution (90 days-2 years). Data is analyzed by relative risk and 95% confidence intervals, and statistically significant numbers are reported.

**Results:**

Prior ACEi use in burn patients increased the relative risk of sustaining AE, ranging between 1.17 and 1.83 when compared to non-ACEi use burn patients, except for re-hospitalization and mortality in the acute phase, with relative risk 0.92 and 0.75, respectively. Prior ACEi use reduced the relative risk of sustaining AE in all time periods, ranging from 0.62-0.95 when compared to the other anti-hypertensive medications except for ARBs. When compared to ARBs, burn patients with prior ACEi use saw increased relative risk of AE ranging from 1.1 to 1.89 through all three time periods.

**Conclusions:**

Prior ACEi use in burn patients is associated with decreased relative risk of sustaining infection, pneumonia, sepsis, re-hospitalization, and mortality when compared to patients with prior beta blocker, calcium channel blocker, or diuretic use. However, it is associated with increased relative risk of AE when compared to patients on prior ARBs therapy or those without prior ACEi therapy.

**Applicability of Research to Practice:**

If patients with prior ACEi use or ARBs use are associated with decreased relative risk of sustaining AE following burn injury, when compared to analogous cohorts, can these medications perform similarly in patients sustaining burn injury without indications due to the modulation of angiotensin II?

**Funding for the study:**

N/A.

